# The Possible Anti-Inflammatory Effect of Dehydrocostus Lactone on DSS-Induced Colitis in Mice

**DOI:** 10.1155/2020/5659738

**Published:** 2020-01-30

**Authors:** Qing Zhou, Wei-xin Zhang, Zong-qi He, Ben-sheng Wu, Zhao-feng Shen, Hong-tao Shang, Tuo Chen, Qiong Wang, Yu-gen Chen, Shu-tang Han

**Affiliations:** ^1^Affiliated Hospital of Nanjing University of Chinese Medicine, Nanjing, Jiangsu, China; ^2^Suzhou Hospital of Traditional Chinese Medicine, Suzhou, Jiangsu, China

## Abstract

**Background:**

Dehydrocostus lactone (DL), one of the main active constituents in *Aucklandia lappa Decne*. (Muxiang), reported to have anti-inflammatory, antiulcer, and immunomodulatory properties. However, the effect of DL on ulcerative colitis (UC) has not been reported. To analyze the anti-inflammatory potential role of DL in UC, we provide a mechanism for the pharmacological action of DL.

**Methods:**

The experimental model of UC was induced by using oral administration of 2% dextran sulfate sodium (DSS) with drinking water in BALB/c mice. Mesalazine (Mes, 0.52 g/kg/d), DL-high doses (DL-H, 20 mg/kg/d), DL-middle doses (DL-M, 15 mg/kg/d), DL-low doses (DL-L, 10 mg/kg/d) were gavaged once a day from day 4 to day 17. Disease activity index (DAI) was calculated daily. On day 18, mice were rapidly dissected and the colorectal tissues were used to detect the levels of UC-related inflammatory cytokines (TNF-*α*, IL-1*β*, MCP-1, MPO, SOD, IL-6, IL-17, and IL-23), IL-6/STAT3 inflammatory signaling pathway (iNOS, COX2, IL-6, GP130, L-17, and IL-23), and colorectal mucosal barrier-related regulatory factors (MUC2, XBP1s, and *α*, IL-1

**Results:**

DL reduced the colorectal inflammation histological assessment, decreased UC-related inflammatory cytokines (TNF-*α*, IL-1*β*, MCP-1, MPO, SOD, IL-6, IL-17, and IL-23), IL-6/STAT3 inflammatory signaling pathway (iNOS, COX2, IL-6, GP130, L-17, and IL-23), and colorectal mucosal barrier-related regulatory factors (MUC2, XBP1s, and *α*, IL-1

**Conclusions:**

DL possessed the potential of anti-inflammatory effect to treated colitis. The protective mechanism of DL may involve in reducing inflammation and improving colorectal barrier function via downregulating the IL-6/STAT3 signaling.

## 1. Introduction

Ulcerative colitis (UC) is a type of inflammatory bowel disease (IBD) which is usually confined to the colon and rectum, with diffuse and superficial lesions of the mucosa [1]. Mucosal inflammation correlated with disease severity is UC characterised [2]. Rich evidences suggested that cytokines played an essential role in the mucosal inflammatory by promoting leukocyte migration to sites of inflammation and ultimately leading to tissue damage and destruction [3]. Also, studies have shown that destruction of MUC2 protein and upregulation of the IL-6/STAT3 inflammatory signaling pathway were the key mechanisms and important targets for the occurrence and development of UC [4].

*Aucklandia lappa Decne*. (Muxiang), a traditional Chinese herbal medicine, first recorded in Sheng Nong's herbal classic, is commonly applied in clinical practice for the treatment of diarrhea from the 2nd century BC in China with good effects and few adverse events. Modern pharmacological studies have demonstrated dehydrocostus lactone (DL), one of the main activating components in *Aucklandia lappa Decne*. (Muxiang), was linked to the regulation of anti-inflammation [5, 6]. To the best of the authors' knowledge, until now, the effect of DL on colitis has not been reported.

Thus, the purpose of this study is to investigate the effect of DL on colitis. In this study, DSS-induced experimental colitis was used. Our results demonstrated that DL possessed the potential of anticolitis efficiency. The protective mechanism of DL may involve in reducing inflammation and improving colorectal barrier function via downregulating the IL-6/STAT3 signaling.

## 2. Materials and Methods

### 2.1. Drugs and Reagents

**Figure 1:**
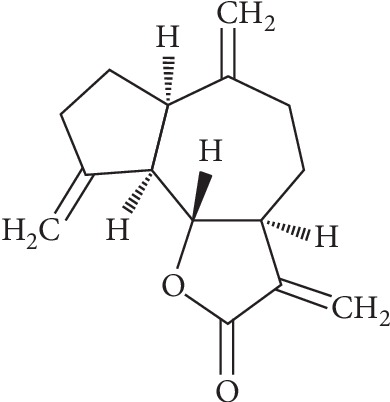
The chemical structures of dehydrocostus lactone.

DL (lot no: Q-016-170816) was purchased from Chengdu Ruifensi Biotechnology Co., Ltd. (Chengdu, China) (Figure 1).The dextran sulfate sodium (DSS) (lot no. Q1723) was purchased from USA MP Ltd. Mesalazine SR Granules (lot no. 160627) was purchased from Shanghai Aizhifa Pharmaceutical Co., Ltd. (Shanghai, China). Stool blood test kit was purchased from Zhuhai Besso Biotechnology Co., Ltd. (Zhuhai, China). TNF-*α*, IL-1*β*, MCP-1, MPO, SOD, IL-6, IL-17, and IL-23 ELISA assay were purchased from Beijing Baiolaibo Technology Co., Ltd. (Beijing, China). iNOS, COX2, IL-6, GP130, STAT3, IL-17, IL-23, MUC2, XBP1s, and *α*-defensins (polymerase chain reaction RT-PCR) were purchased from Shanghai Sangon Biotech (Shanghai, China).

### 2.2. Animals

BALD/c mice (6–8 weeks, 18–22 g) were purchased from the Laboratory Animal Center (Anhui Medical University, China). They were allowed at least 1 week under controlled temperature (22 ± 2°C) and photoperiods (12 h : 12 h light-dark cycle) to adapt to their environment before being used for experiments. There were 60 mice used in the study, 40 in the control group (mesalazine group/Mes, DL-high-dose group/DL-H, DL-middle-dose group/DL-M, DL-low-dose group/DL-L, and each group mice = 10), 10 in the model group, and 10 in the normal group. Care and experimentation of mice were performed in accordance with the guide of laboratory animals (Ministry of Science and Technology of China, 2006) and related ethical regulations of Affiliated Hospital of Nanjing University of Chinese Medicine.

### 2.3. UC Model of DSS-Induced Colitic Mice

Model group, Mes group, DL-H group, DL-M group, and DL-L group had oral administration of 2% DSS via drinking water from day 1 to day 17. Mes (0.52 g/kg/d), DL-H (20 mg/kg/d), DL-M (15 mg/kg/d), and DL-L (10 mg/kg/d) were gavaged once a day from day 4 to day 17. Normal group mice drank water without DSS. Disease activity index (DAI, mice weight, stool consistency, and the presence of gross blood in feces) was calculated once daily. On day 18, mice were executed and rapidly dissected, and the entire colon and rectum were quickly removed.

### 2.4. Histological Assessment of Colon Tissues

On day 18, mice were sacrificed. The colon and rectum segments, from the anus to the appendix, were cut open along the longitudinal axis of the mesentery. Specimens with obvious inflammation/ulcer or specimens 15 cm away from the anus (if a mouse had no obvious lesion) were chosen for histological assessment. This segment of the colon was cut about 0.5 cm, and the paraffin-embedded section was fixed with 4% paraformaldehyde. After HE staining, the pathological changes of the colon were observed under the microscope (magnification *∗* 100/400); the histological lesions of the colon were scored [7] (Table 1). The remaining colon and rectum tissues were used for follow-up studies.

### 2.5. Inflammation Cytokines Determination by ELISA Assay

On day 18, the levels of inflammatory cytokines (TNF-*α*, IL-1*β*, MCP-1, MPO, SOD, IL-6, IL-17, and IL-23) were determined using ELISA kits according to the manufacturer's instructions of Beijing Baiolaibo Technology Co., Ltd. (Beijing, China).

### 2.6. IL-6/STAT3 Inflammatory Signaling Pathway and Colorectal Mucosal Barrier-Related Regulatory Factor Measurement by qRT-PCR

On day 18, the IL-6/STAT3 inflammatory signaling pathway (iNOS, COX2, IL-6, GP130, STAT3, IL-17, and IL-23) and colorectal mucosal barrier-related regulatory factors (MUC2, XBP1s, and *α*-defensins) were evaluated by qRT-PCR in Shanghai Sangon Biotech (Shanghai, China) (Table 2).

### 2.7. Statistical Analysis

All data are shown as mean (M) ± standard error of the mean (S.E.M.), and statistical significance between groups was determined using one-way analysis of variance followed by SPSS software version 15.0. Differences were considered significant at *P* < 0.05.

## 3. Results

### 3.1. DL Attenuated DSS-Induced Chronic Ulcerative Colitis in Mice

**Figure 2:**
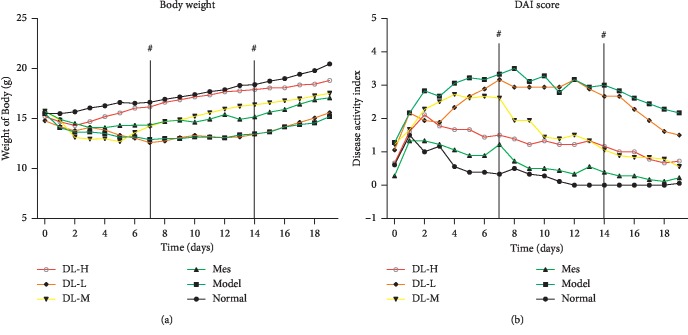
Protective role of DL against DSS-induced colitis in mice. (a) Body weight changes after DSS-induced colitis. (b) Disease activity index (DAI). Animal model of chronic DSS-induced UC in BALB/c mice. ^^*▲*^^*P* < 0.05 and ^*▲▲*^*P* < 0.01, when compared to the model group.

To explore the potential of DL to inhibit inflammation, we tested whether DL had an inhibitory effect on inflammation in DSS-induced colitis mice. We found that treatment of mice with DL delayed the effect on body weight loss and reduced the bloody stools compared to the DSS-treatment group. The DAI score was improved significantly after the treatment, particular to Mes and DL-H groups (Figure 2).

### 3.2. Histological Assessment

Moderate or severity colorectal inflammation, mostly involving the mucosa and submucosa, showing crypt damage, was found in model mice. In the DL-H group and Mes group, the colorectal inflammation score was significantly reduced compared with the model group (Table 3).

### 3.3. DL Reduces Inflammatory Cytokines Expression

**Figure 3:**
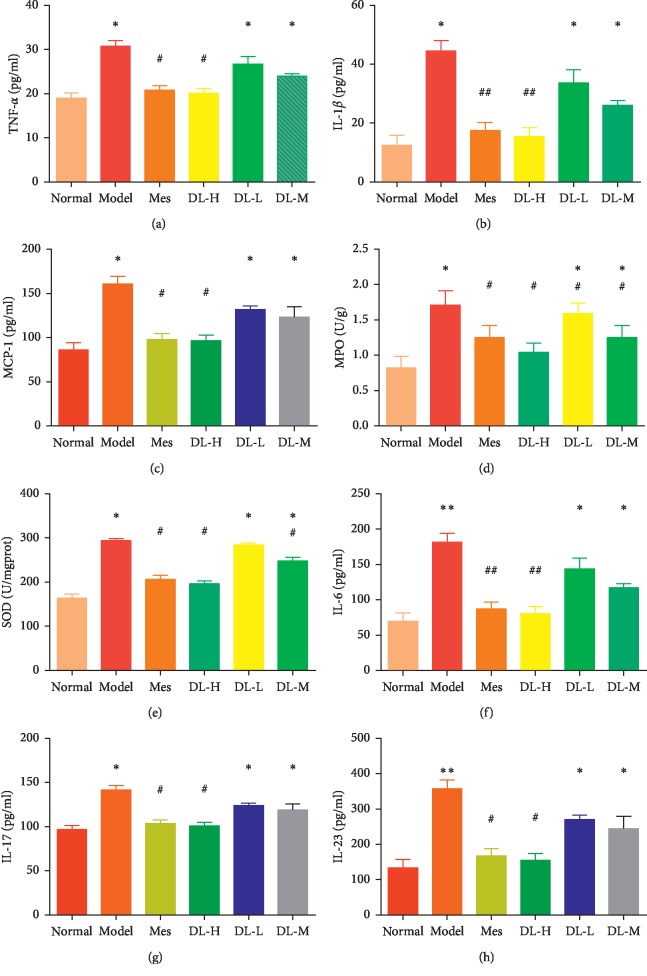
DL reduced inflammatory cytokines expression. (a) TNF-*α*. (b) IL-1*β*. (c) MCP-1. (d) MPO. (e) SOD. (f) IL-6. (g) IL-17. (h) IL-23. Data are presented as the mean ± SD. ^*∗*^*P* < 0.05 and ^*∗∗*^*P* < 0.01, when compared to the normal group; ^*▲*^*P* < 0.05 and ^*▲▲*^*P* < 0.01, when compared to the model group (*n* = 10).

After drinking 2% of DSS, UC related inflammatory cytokines were increased significantly compared with normal group. After medicine intervention, inflammatory cytokines (TNF-*α*, IL-1*β*, MCP-1, MPO, SOD, IL-6, IL-17, IL-23) were decreased, especially in DL-H and Mes group. These results indicated DL could prevent inflammatory reaction through downregulating cytokines expression (Figure 3).

### 3.4. DL Downregulates IL-6/STAT3 Inflammatory Signaling Pathway

**Figure 4:**
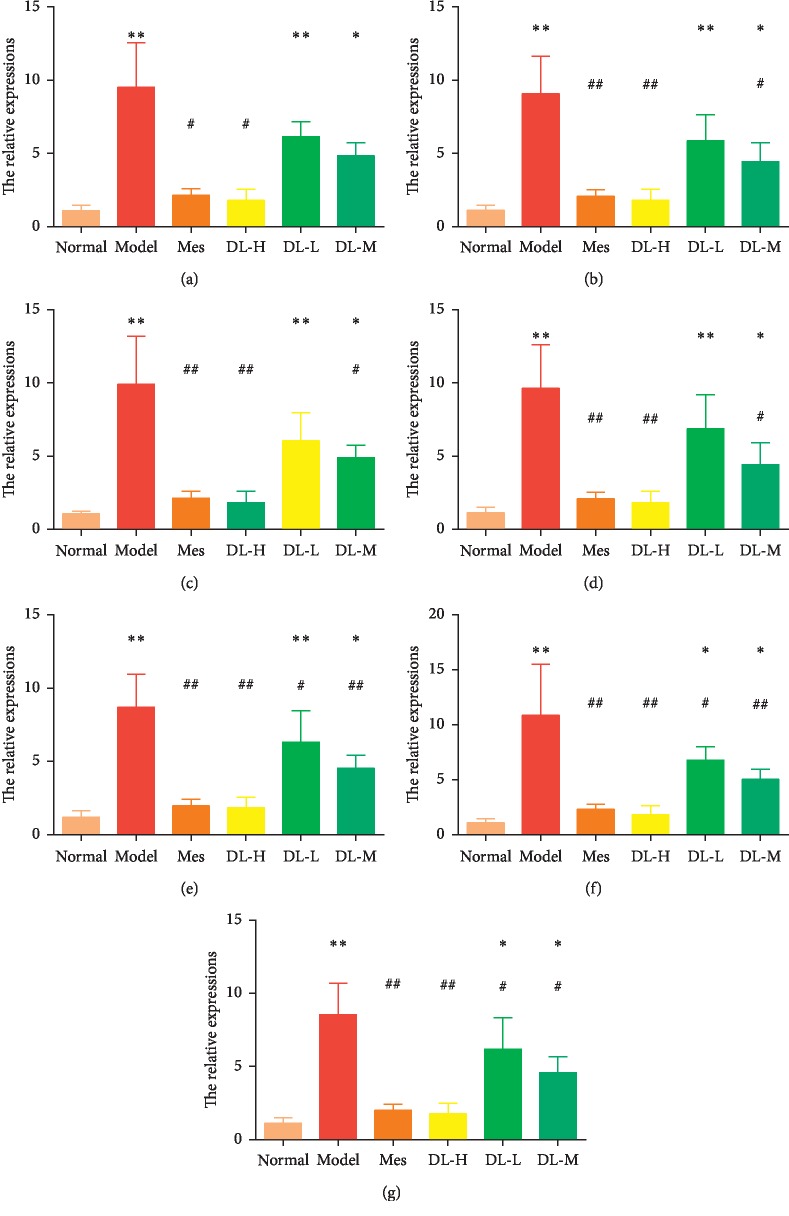
DL downregulated IL-6/STAT3 inflammatory signaling pathway. (a) iNOS. (b) COX2. (c) IL-6. (d) GP130. (e) STAT3. (f) IL-17. (g) IL-23. ^*∗*^*P* < 0.05 and ^*∗∗*^*P* < 0.01, when compared to the normal group; ^*▲*^*P* < 0.05 and ^*▲▲*^*P* < 0.01, when compared to the model group (*n* = 10).

As the important upstream elements of inflammatory cytokines, IL-6/STAT3 inflammatory signaling pathway (iNOS, COX2, IL-6, GP130, IL-17, and IL-23) was measured, respectively. Because of the chemical stimulus, IL-6/STAT3 inflammatory signaling was markedly increased by DSS, but reversed in DL treatment, especially to the DL-H group (Figure 4).

### 3.5. DL Maintains Colorectal Mucosal Barrier Related Regulatory Factors

**Figure 5:**
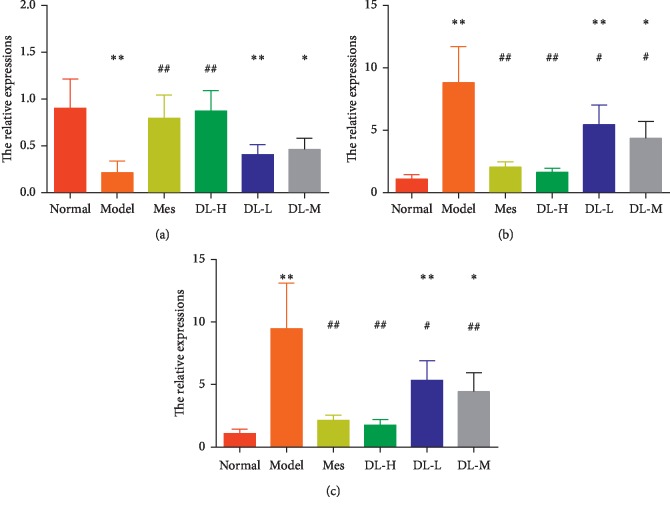
DL maintained colorectal mucosal barrier-related regulatory factors. (a) MUC2. (b) XBP1s. (c) *α*-defensin. ^*∗*^*P* < 0.05 and ^*∗∗*^*P* < 0.01, when compared to the normal group; ^*▲*^*P* < 0.05 and ^*▲▲*^*P* < 0.01, when compared to the model group (*n* = 10).

After drinking 2% of DSS, the key colorectal mucosal barrier protein-MUC2 expression was decreased. The downstream pathway, XBP1s, and *α*-defensin were increased significantly. Compared with the model group, MUC2, XBP1s and *α*-defensin expressions were reversed in the DL group, especially in the DL-H group (Figure 5).

## 4. Discussion

Nowadays, the control of clinical remission was no longer sufficient to the UC patients [8]. Mucosal healing is emerging as a major therapeutic goal, which is associated with a reduced need for surgery and hospitalization [9]. Furthermore, current drugs for UC treatments were unsatisfied as they possessed long-term damage and showed some limitations, such as corticosteroids delayed healing of wounds, induced peptic ulcerations, and suppressed hypothalamic pituitary axis [10]. Thiopurines were associated with nonmelanoma skin cancer [11]. Biologicals and antitumor necrosis factor drugs were associated with both autoimmune disease and malignancies [12].

As an idiopathic, colonic mucosal inflammatory disease, the cause for UC is not fully understood, including genetics, environment, and diet. Of these risks, inflammation appeared to be a key driving factor for the molecular changes in UC. The IL-6/STAT3 inflammatory signaling pathway has been reported to be involved in the pathogenesis and progression of a number of inflammatory disorders. IL-6 signaling through STAT3 was a critical regulator of LPS-driven proinflammatory responses, including TNF-*α*, iNOS, IL-1*β*, IL-6, GP130, IL-17, IL-23, and STAT3 [13–16]. These activation proinflammatory mediators augmented the inflammatory response and damaged the intestinal tissue. Forced IL-6/STAT3 expression was sufficient to drive an increase in endothelial permeability [17].

Mucoprotein2 (MUC2) [18] was a major component of colorectal mucosal protection in mammals, forming a unique protective barrier of the colorectal mucosa to separate colorectal epithelial cells from the external environment, maintaining the homeostasis of colorectal epithelial cells. The reduction of the MUC2 proteins thinned the mucus gel layer, activated *α*-defensin, and led to the endoplasmic reticulum stress reaction [19]. When this reaction surpassed the self-regulatory capacity of the unfolded protein XBP1s reaction, *α*-defensin induced goblet cell morphology change, immatured goblet cell apoptosis, and released proinflammatory mediators, such as IL-6 and IL-1*β*. Activating the IL-6/STAT3 inflammatory signaling pathway also promoted an increase in endothelial monolayer permeability and induced a sustained loss of endothelial barrier function [4, 19–21]. Loss of the protective mucinous membrane resulted in epithelial cell damage and upregulation of the IL-6/STAT3 inflammatory signaling pathway expression in colorectal epithelial cells and finally led to UC [18]. Therefore, the MUC2/IL-6-STAT3 pathway seemed to be a key node in the treatment of UC.

*Aucklandia lappa Decne*. (Muxiang), a traditional Chinese medicine, was first recorded in the book of Shen Nong's herbal classic. The main function of this traditional Chinese medicine was used to treat diarrhea for hundreds of years, which can be summarized as four main functions: invigorating qi, strengthening the spleen, relieving pain, and curing diarrhea. The active components of *Aucklandia lappa Decne*. (Muxiang) were concentrated in its volatile oil and terpenoids, such as dehydrocostus lactone (DL), which was up to 50% of the main active components [5]. DL can improve the inflammatory response and repair colorectal mucositis by downregulating NF-*κ*B and TNF-*α* activation and inhibiting the production of reactive oxygen species [22, 23].

In this study, DL alleviated colorectal damages caused by DSS, ameliorated UC model's body weight fluctuation, reduced the colorectal inflammation histological assessment, decreased UC-related inflammatory cytokines (TNF-*α*, IL-1*β*, MCP-1, MPO, SOD, IL-6, IL-17, and IL-23), downregulated the IL-6/STAT3 inflammatory signaling pathway (iNOS, COX2, IL-6, GP130, STAT3, IL-17, and IL-23), repaired the key colorectal mucosal barrier protein-MUC2, and inhibited the downstream pathway (XBP1s and *α*-defensin), suggesting that the protective mechanism of DL for UC may involve in repairing MUC2 and downregulating the IL-6/STAT3 inflammatory signaling pathway.

## 5. Conclusion

Our study explored the potential role of DL in anti-inflammation for UC and provided a mechanism for the pharmacological action of DL. The mechanism of DL might involve in repairing the key colorectal mucosal barrier protein-MUC2 via downregulating the IL-6/STAT3 inflammatory signaling pathway. Our results suggested that DL might be a promising candidate for the treatment of UC.

## Figures and Tables

**Table 1:** Colon histological lesion score.

Score	Criterion
0	Normal tissue
1	Superficial epithelial damage
2	Focal ulcer confined to the mucosa
3	Focal transmural infammation and ulceration
4	Extensive transmural ulcer and infammation, normal mucosa between lesions
5	Extensive flaky transmural ulcers and infammation

**Table 2:** Primers and amplification conditions used for RT-PCR in BALB/c mice.

Primers	Primer sequences	
	Sense	Antisense
iNOS	5′-CCCTTCCGAAGTTTCTGGCAGCA-3′	5′-GGCTGTCAGAGCCTCGTGGCTTT-3′
COX2	5′-CACTACATCCTGACCCACTT-3′	5′-ATGCTCCTGCTTGAGTATGT-3′
IL-6	5′-GGCGGATCGGATGTTGTGAT-3′	5′-GGACCCCAGACAATCGGTTG-3′
GP130	5′-CCGTGTGGTTACATCTACCCT-3′	5′-CGTGGTTCTGTTGATGACAGTG-3′
STAT3	5′-CAATACCATTGACCTGCCGAT-3′	5′-GAGCGACTCAAACTGCCCT-3′
IL-17	5′-TGAAAACACAGAAGTAACGTCC-3′	5′-CCCAGGAGGAAATTGTAATGGG-3′
IL-23	5′-ATGCTGGATTGCAGAGCAGTA-3′	5′-ACGGGGCACATTATTTTTAGTCT-3′
MUC2	5′-TCCAGGTCTCGACATTAGCAG-3′	5′-GTGCTGAGAGTTTGCGTGTCT-3′
XBP1s	5′-GTGGACCAGTTAAGCATGAGG-3′	5′-GCTCTCGGCGCTTGTTGAT-3′
*α*-defensins	5′-GAATCCGAGAGGATAAGGACCA-3′	5′-TCCATTGAAAGGGCAATAGGGA-3′

**Table 3:** Histological assessment.

Group	Number (*n*)	Pathology score
Normal	10	0
Model	10	4.33 ± 0.52^*∗∗▲▲*^
Mes	10	0.62 ± 0.55^*∗▲▲*^
DL-L	10	1.66 ± 0.82^*∗∗▲▲*^
DL-M	10	1.06 ± 0.40^*∗∗▲▲*^
DL-H	10	0.75 ± 0.32^*▲▲*^

^*∗*^ *P* < 0.05 and ^*∗∗*^*P* < 0.01, when compared to the normal group; ^*▲*^*P* < 0.05 and ^*▲▲*^*P* < 0.01, when compared to the model group.

## Data Availability

The research methods, research results, and the processed data are available in Tables 1–3 and Figures 1–4 in our manuscript.

## Abbreviations

DL: Dehydrocostus lactone
UC: Ulcerative colitis
IBD: Inflammatory bowel disease
DSS: Dextran sulfate sodium
Mes: Mesalazine group
DL-H: DL-high-dose group
DL-M: DL-middle-dose group
DL-L: DL-low-dose group
DAI: Disease activity index
MUC2: Mucoprotein2.
